# Supplementary material for: Detection of SARS-CoV-2 RNA in serum is associated with increased mortality risk in hospitalized COVID-19 patients
Source: Sci Rep. 2021 Jun 23;11:13134. doi: 10.1038/s41598-021-92497-1 (PMC8222315; doi:10.1038/s41598-021-92497-1)
Supplement: Supplementary file 1 — Supplementary Information. [file 41598_2021_92497_MOESM1_ESM.pdf]

SUPPLEMENTARY MATERIAL

FULL TITLE: Detection of SARS-CoV-2 RNA in serum is associated with increased mortality risk in hospitalized COVID-19 patients.

SHORT TITLE: Increased mortality in COVID-19 patients with relevant viremia.

AUTHORS: Diego A. Rodríguez-Serrano[1¶*], Emilia Roy-Vallejo[2¶], Nelly D. Zurita Cruz[3], Alexandra Martín Ramírez[3], Sebastián C. Rodríguez-García[4], Nuria Arevalillo-Fernández[1], José María Galván-Román[2], Leticia Fontán García-Rodrigo[3], Lorena Vega-Piris[5], Marta Chicot Llano[1], David Arribas Méndez[6], Begoña González de Marcos[1], Julia Hernando Santos[6], Ana Sánchez Azofra[7], Elena Ávalos Pérez-Urria[7], Pablo Rodriguez-Cortes[2], Laura Esparcia[8], Ana Marcos-Jimenez[8], Santiago Sánchez-Alonso[8], Irene Llorente[4], Joan Soriano[7,9], Carmen Suárez Fernández[2,9], Rosario García-Vicuña[4], Julio Ancochea[7,9], Jesús Sanz[2], Cecilia Muñoz-Calleja[8,9], Rafael de la Cámara[10], Alfonso Canabal Berlanga[1], Isidoro González-Álvaro[4&], Laura Cardeñoso[3&], on behalf of the REINMUN-COVID Group[^].

[¶] These authors have contributed equally.

[&] These authors also contributed equally to this work.

* Corresponding author: cancabrilla@hotmail.com (DA R-S)

AFFILIATIONS

[1] Intensive Care Unit. Hospital Universitario La Princesa. Madrid. Spain

[2] Internal Medicine Department. Hospital Universitario La Princesa. IIS-IP. Madrid. Spain

[3] Microbiology Department. Hospital Universitario La Princesa. IIS-IP. Madrid. Spain

[4] Rheumatology Department. Hospital Universitario La Princesa. IIS-IP. Madrid. Spain

[5] Methodology Unit. Biomedical Research Institute. Hospital Universitario La Princesa. IIS-IP. Madrid. Spain

[6] Anaesthesiology Department. Hospital Universitario La Princesa. IIS-IP. Madrid. Spain

[7] Pneumology Department. Hospital Universitario La Princesa. IIS-IP. Madrid. Spain

[8] Immunology Department. Hospital Universitario La Princesa. IIS-IP. Madrid. Spain

[9] Universidad Autónoma de Madrid. Madrid, Spain.

[10]Hematology Department. Hospital Universitario La Princesa. IIS-IP. Madrid. Spain

^ Membership of REINMUN-COVID Group is provided in the Acknowledgments.

S1 Table. Baseline clinical characteristics of patients according to SARS-CoV-2 RNA detection in blood by Thermo Fisher Systems technique.

	Study Population (n = 193)	Viremia Absent (n = 78)	Viremia Present (n = 115)	P value
**Age**	**63 (55 - 71)**	**61 (53 - 69)**	**65 (59 - 71)**	**0.012**
Male sex	134 (69)	50 (64)	84 (73)	0.186
Comorbidities	137 (71)	55 (70.5)	82 (71)	0.905
Duration of symptoms at admission (days)	6 (4 - 8)	7 (4-9)	6 (5-8)	0.989
**Baseline PaO_2/FiO_2**	**188 (100 - 282)**	**255 (172 – 352)**	**146 (87 – 232)**	**< 0.001**
Treatment during hospitalization				
Hydroxychloroquine	186 (96)	76 (97)	110 (96)	0.515
Lopinavir/Ritonavir	168 (87)	64 (82)	104 (90)	0.089
Azithromycin	133 (69)	54 (69)	79 (69)	0.937
Interferon-β	8 (4)	2 (3)	6 (5)	0.364
**Glucocorticoids**	**134 (69)**	**48 (62)**	**86 (75)**	**0.050**
**Methylprednisolone bolus**	**101 (63)**	**30 (51)**	**71 (70)**	**0.018**
**Tocilizumab**	**91 (47)**	**24 (31)**	**67 (58)**	**< 0.001**
Laboratory Findings				
WBC ($10^3/mm^3$) NR: 4.00 - 10.00	7.18 (4.82 – 9.61)	6.91 (4.89 – 8.56)	7.45 (4.66 – 10.89)	0.328
**Lymphocyte/mm^3** NR: 1.00 -4.00	**790 (535 – 1165)**	**1070 (755 – 1485)**	**700 (535 – 930)**	**< 0.001**
**Creatinine (mg/dl)** NR:0.70 - 1.20	**0.86 (0.73 – 1.09)**	**0.83 (0.67 – 1.01)**	**0.92 (0.73 – 1.16)**	**0.044**
**LDH (U/L)** NR: 135 - 225	**390 (278 - 512)**	**289 (223 - 390)**	**457 (350 – 548)**	**< 0.001**
CK (U/L) NR: 20 - 180	100 (49 - 270)	93 (58-155)	117 (48 - 293)	0.412
**Serum IL-6 (pg/ml)** NR: < 30 pg/ml	**20.7 (7.9 – 52.1)**	**17.6 (7.5 – 30.4)**	**26.5 (8.2 – 75.0)**	**0.012**
Ferritin (ng/ml) NR: 30 - 400	1542 (871 – 2617)	1390 (634 - 2068)	1637 (944 - 3214)	0.104
**CRP (mg/dL)** NR: 0.00 - 0.50	**12.2 (5.5 – 23.0)**	**9.1 (4.04 – 18.7)**	**14.1 (7.3 – 25.8)**	**0.002**
**PCT (ng/ml)** NR: 0.05 - 0.09	**0.20 (0.11 – 0.41)**	**0.13 (0.07 – 0.36)**	**0.23 (0.13 – 0.46)**	**0.015**
**D-dimer (mg/ml)** NR:0.14 - 0.50	**0.78 (0.52 – 1.47)**	**0.70 (0.39 – 1.08)**	**0.87 (0.62 – 2.02)**	**0.004**

All categorical variables are expressed as number (%) and quantitative variables as median (p25-75). PaO2/FiO2: arterial oxygen tension – fraction of inspired oxygen ratio; WBC: White Blood Count; NR: Normal Range; LDH: Lactate Dehydrogenase; CK: Creatin Kinase; IL6: Interleukin-6; CRP: C-Reactive Protein; PCT: Procalcitonin.

S2 Table. Multiorgan failure and dysfunction syndrome according to baseline SARS-CoV-2 RNA detection in serum (Thermo Fisher Scientific).

	Negative Viremia (n = 26)	Positive Viremia (n = 60)	Total (n = 86)	P value
**MODS (pa)**	12 (46.2)	26 (43.3)	38 (44.2)	0.983
**MODS (24 hrs)**	19 (73.1)	40 (66.7)	59 (68.6)	0.832
**MOF (pa)**	1 (3.8)	3 (5.0)	4 (4.7)	> 0.99
**MOF (24 hrs)**	13 (50)	29 (48.3)	42 (48.8)	0.922
**Cardiovascular (pa)**	1 (3.8)	5 (8.3)	6 (7.0)	0.661
**Liver (pa)**	3 (11.5)	4 (6.7)	7 (8.1)	0.674
**Renal (pa)**	5 (19.2)	9 (15.0)	14 (16.3)	0.758
**CNS (pa)**	2 (7.7)	6 (10.0	8 (9.3)	> 0.99
**Coagulation (pa)**	4 (15.4)	12 (20.0)	16 (18.6)	0.767
**Cardiovascular (24 hrs)**	14 (50.0)	33 (55.0)	47 (54.7)	0.719
**Liver (24 hrs)**	2 (7.7)	6 (10.0)	8 (9.3)	> 0.99
**Renal (24 hrs)**	4 (15.4)	9 (15.0)	13 (15.1)	> 0.99
**CNS (24 hrs)**	2 (7.7)	3 (5.0)	5 (5.8)	0.648
**Coagulation (24 hrs)**	6 (23.1)	9 (15.0)	15 (17.4)	0.435

MODS (pa): Multiorgan Disfunction syndrome prior to admission; MODS (24 hrs): Multiorgan Disfunction syndrome during the first 24 hours at the ICU; MOF (pa): Multiorgan Failure prior to admission; MOF (24 hrs): Multiorgan Failure during the first 24 hours at the ICU. Cardiovascular (pa): cardiovascular dysfunction/failure prior to admission. Liver (pa): liver dysfunction/failure prior to admission; Renal (pa): renal dysfunction/failure prior to admission; CNS (pa): central nervous system dysfunction/failure prior to admission; Coagulation (pa): platelets dysfunction/failure prior to admission; Cardiovascular (24 hrs): cardiovascular dysfunction/failure during the first 24 hours at the ICU**;** Liver (24 hrs): dysfunction/failure during the first 24 hours at the ICU; Renal (24 hrs): dysfunction/failure during the first 24 hours at the ICU; CNS (24 hrs): central nervous system dysfunction/failure during the first 24 hours at the ICU; Coagulation (24 hrs): platelets dysfunction/failure during the first 24 hours at the ICU.

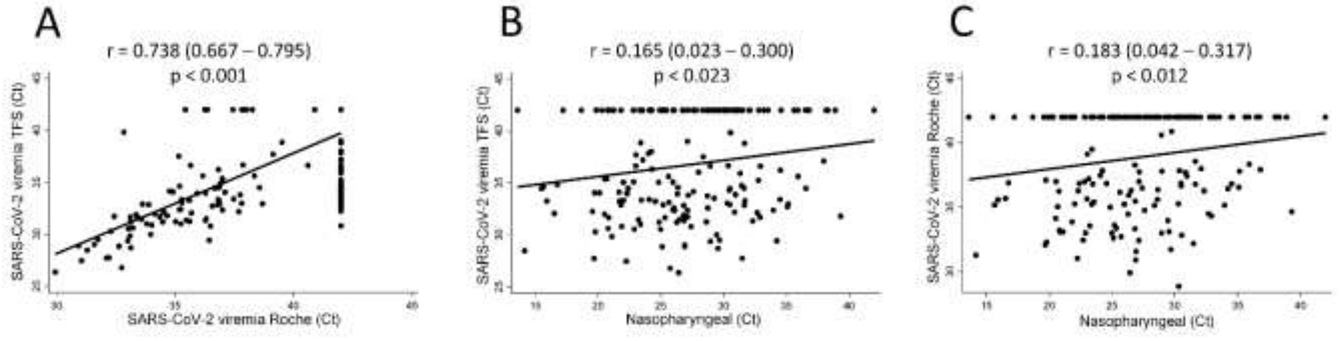

S1 Figure: Correlation between quantitative detection of SARS-CoV-2 in serum (viremia), either with Thermo Fisher Scientific (TFS) or Roche, and in nasopharyngeal and throat swab (NPTS). A) Correlation between viremia with TFS and Roche techniques; Correlation between SARS-CoV-2 detection in NPTS samples and viremia using the TFS technique (B) or the Roche technique (C). Data are shown as dot plots of the mean Ct values of the RT-PCR for SARS-CoV-2 and the fitted linear prediction obtained with the option *lfit* of the command *twoway* of Stata. Correlation coefficients and significance levels were estimated with the Pearson's test.

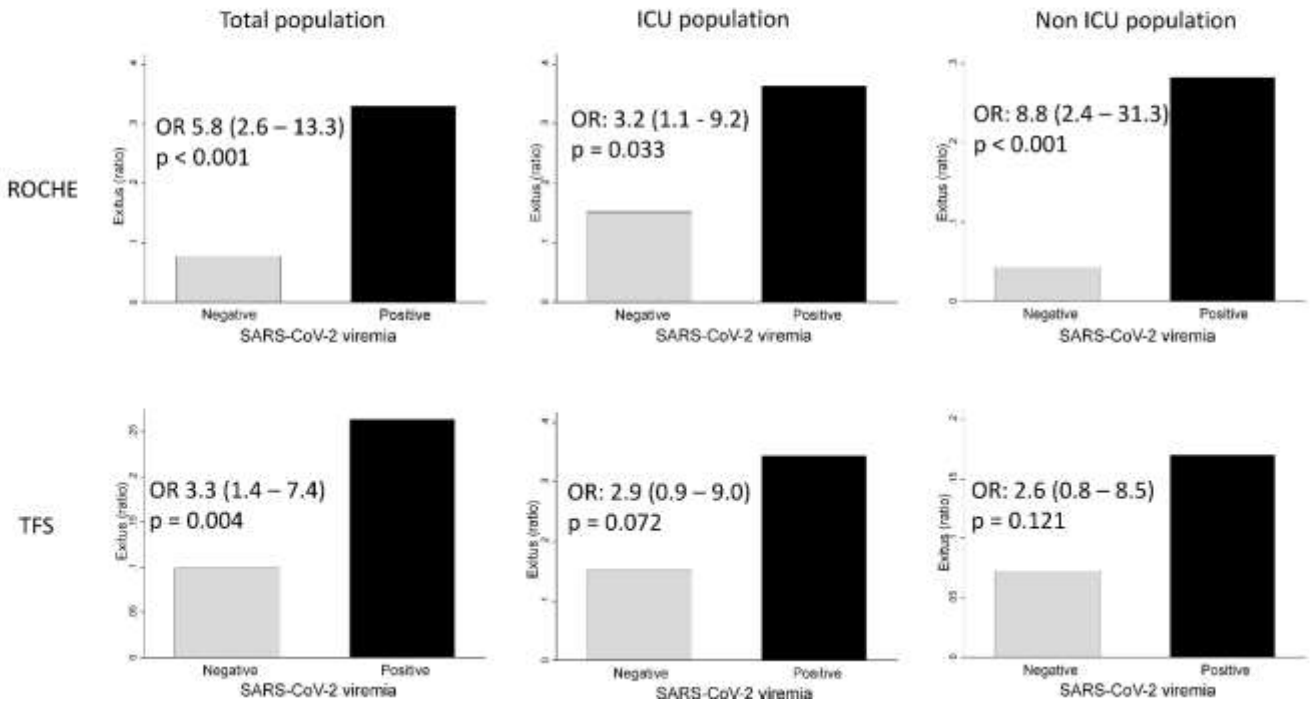

S2 Figure. Mortality is higher among patients with positive viremia, either in the whole population (left panels), in those patients requiring ICU admission (middle panels) or those no requiring ICU (right panels). Odds ratio (OR) and significance level for mortality according to the presence of viremia was estimated with the *cs* command of Stata. TFS: Thermo Fisher Scientific.

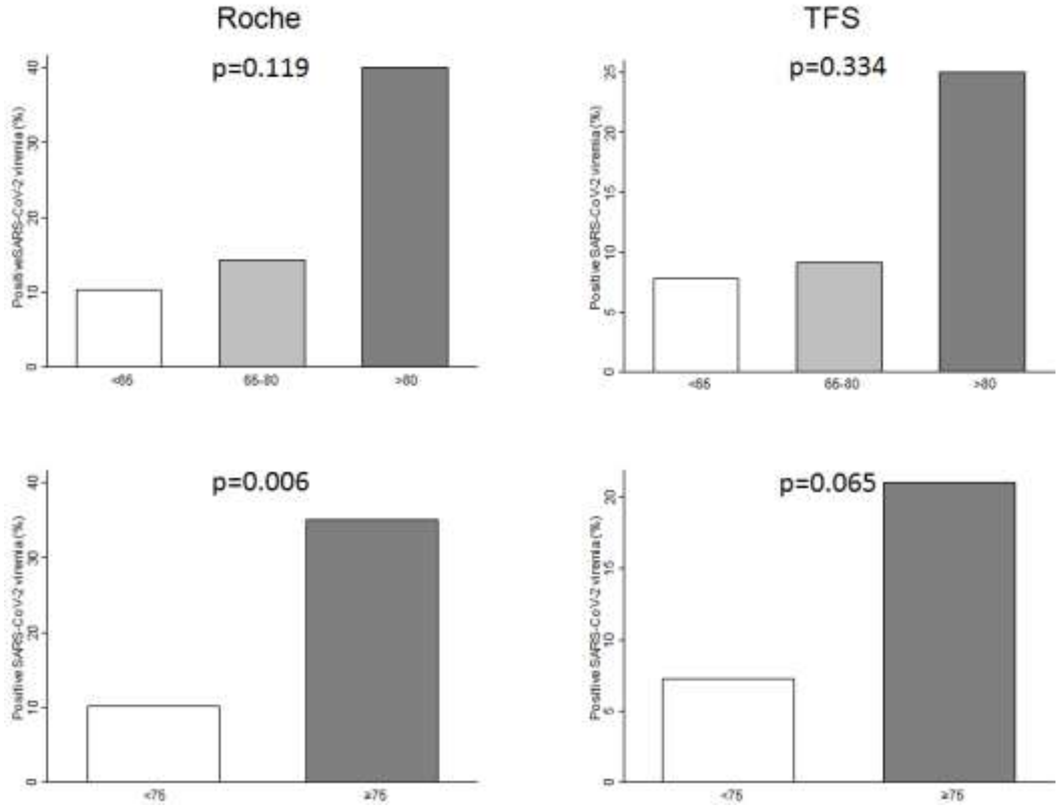

S3 Figure. Percentage of positive patients for relevant SARS-CoV-2 viremia by age group and RT-PCR technique. Upper panels: patients were classified in age groups as <65 years, 65-80 years and >80 years. Lower panels: patients were clustered in age groups as <75 years old and >75 years old. Results with Roche technique are shown on the left side and those with Thermo Fisher Scientific technique (TFS) on the right side. Significance levels were estimated with the Fisher's exact test.

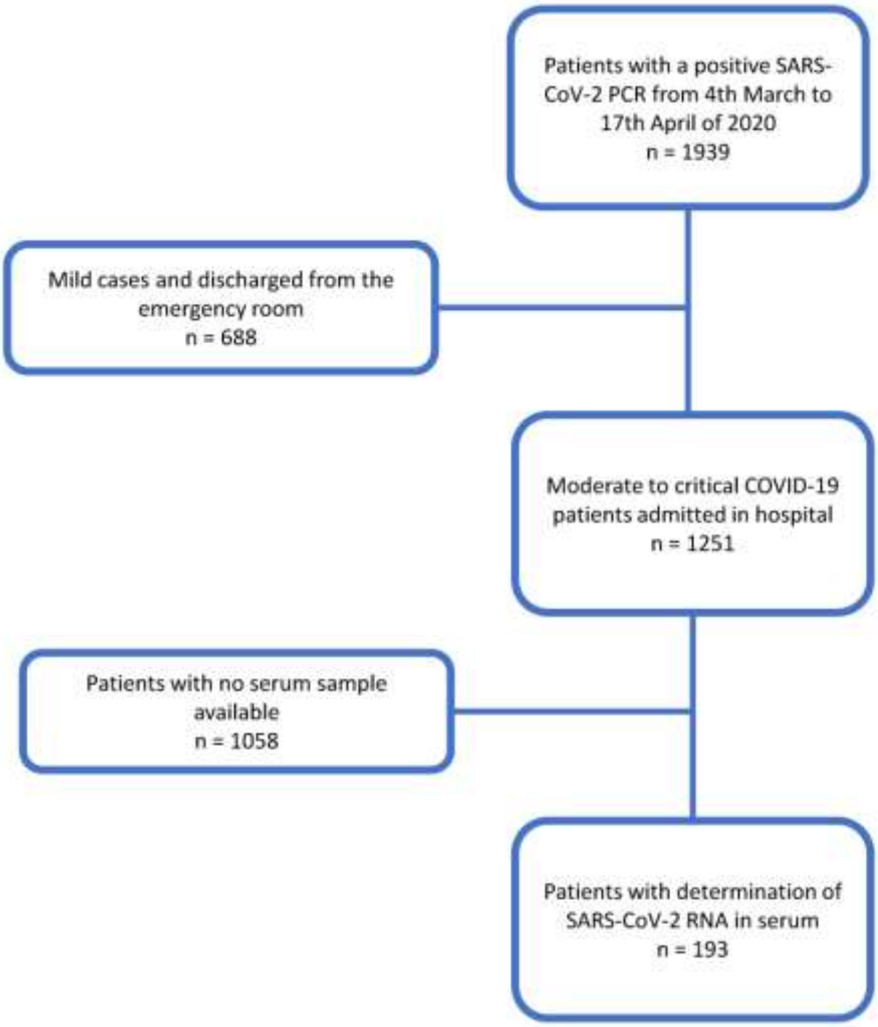

S4 Figure: Flowchart of patients included in the study.

STROBE Statement—checklist of items that should be included in reports of observational studies

	Item No	Recommendation	Page No
**Title and abstract**	1	(*a*) Indicate the study's design with a commonly used term in the title or the abstract	1
		(*b*) Provide in the abstract an informative and balanced summary of what was done and what was found	3-4
**Introduction**			
Background/rationale	2	Explain the scientific background and rationale for the investigation being reported	5-6
Objectives	3	State specific objectives, including any prespecified hypotheses	6
**Methods**			
Study design	4	Present key elements of study design early in the paper	13
Setting	5	Describe the setting, locations, and relevant dates, includi,ng periods of recruitment, exposure, follow-up, and data collection	13-14
Participants	6	(*a*) *Cohort study*—Give the eligibility criteria, and the sources and methods of selection of participants. Describe methods of follow-up<br>*Case-control study*—Give the eligibility criteria, and the sources and methods of case ascertainment and control selection. Give the rationale for the choice of cases and controls<br>*Cross-sectional study*—Give the eligibility criteria, and the sources and methods of selection of participants	13-14
		(*b*) *Cohort study*—For matched studies, give matching criteria and number of exposed and unexposed<br>*Case-control study*—For matched studies, give matching criteria and the number of controls per case	
Variables	7	Clearly define all outcomes, exposures, predictors, potential confounders, and effect modifiers. Give diagnostic criteria, if applicable	16-17
Data sources/ measurement	8*	For each variable of interest, give sources of data and details of methods of assessment (measurement). Describe comparability of assessment methods if there is more than one group	*14-17*
Bias	9	Describe any efforts to address potential sources of bias	
Study size	10	Explain how the study size was arrived at	S1 Fig
Quantitative variables	11	Explain how quantitative variables were handled in the analyses. If applicable, describe which groupings were chosen and why	16-17
Statistical methods	12	(*a*) Describe all statistical methods, including those used to control for confounding	17-19
		(*b*) Describe any methods used to examine subgroups and interactions	17-18
		(*c*) Explain how missing data were addressed	
		(*d*) *Cohort study*—If applicable, explain how loss to follow-up was addressed<br>*Case-control study*—If applicable, explain how matching of cases and controls was addressed<br>*Cross-sectional study*—If applicable, describe analytical methods taking account of sampling strategy	

		(*e*) Describe any sensitivity analyses	

Results

Participants	13*	(a) Report numbers of individuals at each stage of study—eg numbers potentially eligible, examined for eligibility, confirmed eligible, included in the study, completing follow-up, and analysed	S1 Fig
		(b) Give reasons for non-participation at each stage	S1 Fig
		(c) Consider use of a flow diagram	S1 Fig
Descriptive data	14*	(a) Give characteristics of study participants (eg demographic, clinical, social) and information on exposures and potential confounders	6-8
		(b) Indicate number of participants with missing data for each variable of interest	
		(c) *Cohort study*—Summarise follow-up time (eg, average and total amount)	
Outcome data	15*	*Cohort study*—Report numbers of outcome events or summary measures over time	
		Case-control study—Report numbers in each exposure category, or summary measures of exposure	
		Cross-sectional study—Report numbers of outcome events or summary measures	*Fig.1, S3 Fig, Fig. 2*
Main results	16	(*a*) Give unadjusted estimates and, if applicable, confounder-adjusted estimates and their precision (eg, 95% confidence interval). Make clear which confounders were adjusted for and why they were included	6-10
		(*b*) Report category boundaries when continuous variables were categorized	16-17
		(*c*) If relevant, consider translating estimates of relative risk into absolute risk for a meaningful time period	9-10
Other analyses	17	Report other analyses done—eg analyses of subgroups and interactions, and sensitivity analyses	

Discussion

Key results	18	Summarise key results with reference to study objectives	10-12
Limitations	19	Discuss limitations of the study, taking into account sources of potential bias or imprecision. Discuss both direction and magnitude of any potential bias	12-13
Interpretation	20	Give a cautious overall interpretation of results considering objectives, limitations, multiplicity of analyses, results from similar studies, and other relevant evidence	10-12
Generalisability	21	Discuss the generalisability (external validity) of the study results	10-13

Other information

Funding	22	Give the source of funding and the role of the funders for the present study and, if applicable, for the original study on which the present article is based	Submission System

*Give information separately for cases and controls in case-control studies and, if applicable, for exposed and unexposed groups in cohort and cross-sectional studies.

Note: An Explanation and Elaboration article discusses each checklist item and gives methodological background and published examples of transparent reporting. The STROBE checklist is best used in conjunction with this article (freely available on the Web sites of PLoS Medicine at http://www.plosmedicine.org/, Annals of Internal Medicine at http://www.annals.org/, and Epidemiology at http://www.epidem.com/). Information on the STROBE Initiative is available at www.strobe-statement.org.
